# Supplementary material for: Identification of Stable Meta-QTLs and Candidate Genes Underlying Fiber Quality and Agronomic Traits in Cotton
Source: Plants (Basel). 2025 Oct 24;14(21):3252. doi: 10.3390/plants14213252 (PMC12609637; doi:10.3390/plants14213252)
Supplement: Supplementary file 1 [file plants-14-03252-s001.zip › plants-3862712-supplementary.pdf]

Table S1. Detailed distribution of quantitative trait loci (QTL) for fiber quality, yield and morpho-biological traits across chromosomes.

Trait/chr	A01	A02	A03	A04	A05	A06	A07	A08	A09	A10	A11	A12	A13	D01	D02	D03	D04	D05	D06	D07	D08	D09	D10	D11	D12	D13
QTLs associated with fiber quality																										
FL	6	9	11	6	21	2	10	6	4	13	7	7	4	17	7	8	5	12	11	7	12	3	10	3	13	8
FE	4	3	3	5	7	0	5	3	3	5	5	4	4	11	7	5	0	8	7	5	3	1	1	11	9	3
FM	8	0	7	2	10	3	10	4	5	8	1	3	1	14	5	16	10	4	10	5	7	2	10	20	17	9
FS	8	6	8	9	17	6	41	6	14	8	14	4	11	18	1	8	16	8	9	17	13	10	7	12	23	3
FU	4	4	1	3	11	2	5	5	22	2	7	6	3	11	2	5	5	6	8	11	2	1	7	8	3	6
FMAT	0	2	0	0	0	2	2	0	0	1	0	0	0	3	0	0	4	0	0	0	4	0	0	0	2	0
FSFI	0	0	0	0	0	0	0	0	0	0	0	0	0	0	0	0	0	0	0	0	0	0	1	0	2	0
FSCI	0	0	0	0	0	0	0	0	2	0	0	0	0	0	0	0	2	0	0	0	0	0	0	0	0	0
FB	0	0	0	0	1	0	1	0	0	0	1	0	0	1	0	0	0	0	0	0	0	1	1	0	0	0
FR	0	0	0	0	0	0	0	0	1	1	0	0	0	2	0	0	0	0	0	0	0	2	2	0	0	0
FUHML	0	0	0	2	0	0	2	0	0	0	0	0	0	2	2	0	0	2	0	2	0	0	0	0	0	0
QTLs associated with yield traits																										
SCY	0	0	1	0	0	0	6	0	2	3	0	0	2	1	0	0	0	1	2	1	0	4	0	3	1	6
SCW	0	0	2	0	0	0	0	4	4	6	0	0	0	0	0	2	0	4	4	2	0	0	6	6	2	4
SY	0	0	1	0	0	0	2	0	2	0	0	0	0	0	0	1	1	0	0	1	0	0	0	1	0	0
SI	0	0	8	0	5	4	8	2	6	1	2	1	1	3	4	0	5	4	5	5	1	7	3	5	4	1
LY	0	0	1	0	0	0	1	1	1	0	0	0	1	1	0	1	1	1	1	1	0	0	0	5	0	1
LP	0	1	17	4	9	2	2	1	3	5	2	7	4	7	2	4	2	0	7	5	5	4	3	1	4	4
LI	1	0	4	1	0	4	0	4	0	1	0	1	1	2	1	2	3	0	1	1	1	0	4	0	2	0
BW	2	0	4	1	7	2	0	1	4	1	1	2	2	4	2	0	4	11	4	4	7	1	1	2	3	2
BN	0	0	1	0	0	0	0	0	0	0	0	0	0	0	0	1	2	2	3	6	2	0	0	3	0	0
LW	0	0	0	0	0	0	0	0	0	0	0	0	0	0	1	0	0	0	0	0	0	0	0	0	0	0
HSW	0	0	0	0	0	2	0	0	0	1	0	0	0	0	0	0	0	0	0	0	0	1	0	0	0	0
PB	0	0	0	0	0	0	0	0	0	0	1	0	1	0	0	0	0	0	0	0	0	0	0	0	0	0
NOB	0	0	0	0	0	0	0	0	0	0	0	0	0	0	0	0	0	0	0	0	0	0	0	0	0	1
FN	0	0	0	0	0	0	0	0	0	0	0	0	0	0	0	0	0	0	0	0	0	0	0	1	0	0
NB	0	0	0	1	0	1	0	0	0	1	0	0	0	0	0	0	0	0	0	0	0	0	0	0	0	0
SW	0	0	0	0	0	0	0	1	0	0	0	0	0	0	0	0	0	1	0	0	0	0	0	0	0	0

Fbbw	0	0	0	0	0	0	0	0	0	0	0	0	0	0	0	2	0	0	0	0	0	0	0	0	0	0
QTLs associated with morphobiological traits																										
PH	7	1	3	0	14	1	3	0	4	7	7	4	8	4	6	0	33	1	14	10	1	0	4	0	10	13
NFB	0	0	0	0	0	0	0	0	0	0	0	0	0	0	1	2	0	0	0	0	1	0	0	0	1	0
NFFB	1	2	8	3	2	0	7	0	1	0	4	0	8	4	13	1	23	5	0	2	4	1	3	10	0	1
TNB	0	0	0	0	0	0	0	0	0	0	0	0	0	0	0	0	0	1	0	0	0	0	0	0	0	0
TNN	0	0	0	0	0	0	0	0	0	0	0	0	0	0	0	0	0	0	0	0	0	0	1	0	0	0
TNBM	0	0	0	0	0	0	0	0	0	0	0	2	0	0	2	0	0	4	0	0	2	0	0	2	0	0
FBN	2	0	0	3	2	1	2	1	0	0	0	1	0	3	0	0	0	2	1	2	4	1	0	1	0	2
FT	0	7	6	0	1	0	3	2	2	4	4	2	0	1	4	0	11	0	1	0	0	1	0	1	0	2
FBP	1	0	2	1	3	0	0	2	2	1	0	4	0	4	0	9	0	4	3	2	0	0	4	2	1	1
FSH	0	0	0	0	0	0	0	0	0	0	0	0	0	0	2	0	0	0	0	0	0	0	0	0	0	0
TNSB	0	0	2	0	1	0	0	2	0	0	0	0	2	0	0	0	0	0	6	2	0	0	0	0	0	0
SH	0	0	0	0	0	0	0	0	0	0	0	0	0	0	1	0	0	0	0	0	0	0	0	1	0	0
RL	0	0	0	0	1	0	0	0	0	0	0	0	3	1	0	1	0	0	0	1	0	1	0	2	0	0
RSA	0	0	0	0	1	0	0	0	0	0	0	0	4	0	0	0	0	0	1	1	0	0	0	0	0	0
RV	0	0	0	0	1	0	0	0	0	0	0	0	3	0	0	0	0	0	2	1	0	0	0	1	0	0
NRT	0	0	0	0	0	0	0	0	0	0	0	0	1	0	0	0	0	0	1	0	0	0	0	0	0	0
NRF	0	0	0	0	0	0	0	0	0	0	0	0	1	1	0	0	0	0	1	0	2	0	0	0	0	0
RFW	0	0	0	0	0	0	0	0	0	0	0	0	0	0	1	0	0	0	0	0	0	0	1	0	0	0
LOB	0	1	0	0	0	0	0	0	0	0	0	0	0	0	0	1	0	0	1	0	0	0	0	0	0	0
LOP	0	1	0	0	0	0	0	0	0	0	0	0	0	0	0	0	0	0	0	0	0	0	0	0	0	0
SLA	0	0	0	0	0	0	0	0	1	0	0	0	0	0	0	0	0	0	0	0	0	0	0	0	0	0
LFMP	0	0	0	0	0	0	1	0	0	0	0	1	0	0	0	0	0	0	0	0	0	0	0	0	0	0
STLH	0	0	0	0	0	0	0	0	0	0	0	0	0	0	1	0	0	0	1	0	0	0	0	0	0	0
Bla	0	0	0	0	2	0	0	0	0	0	0	0	0	0	0	0	0	0	0	0	0	0	0	0	0	0
PBS	0	0	0	0	0	0	0	0	0	0	0	0	0	0	0	0	0	0	0	0	0	0	0	1	0	0
SFW	0	0	0	0	0	0	0	0	0	0	0	0	0	0	1	0	0	0	0	0	0	0	0	0	0	0
SDW	0	0	0	0	0	0	0	0	1	0	0	0	0	0	1	0	0	0	0	0	0	0	0	0	0	1
LOS	0	0	0	0	0	0	0	0	0	0	0	0	0	0	0	0	0	3	0	0	0	0	0	0	0	0
QTLs associated with physiological and biochemical traits																										
CP	1	0	0	0	0	1	1	0	0	0	1	1	0	1	1	0	0	0	0	0	1	1	1	0	0	1

CO	0	1	0	0	0	1	1	1	0	0	0	1	0	1	1	1	0	1	1	0	0	1	1	0	0	1
PA	1	1	0	0	0	1	0	0	0	1	0	1	0	0	2	1	0	0	1	0	0	1	1	0	0	0
LA	0	0	0	1	0	0	0	0	0	0	0	0	1	1	0	1	0	1	1	0	0	0	0	1	0	0
SA	0	0	0	0	2	1	3	0	0	0	0	2	0	2	1	0	0	0	0	0	2	1	0	1	0	0
OA	0	0	0	0	0	1	1	1	0	1	1	1	1	0	0	0	0	1	1	0	0	0	0	1	0	0
Pro	0	0	0	1	0	0	0	0	0	0	0	0	0	0	0	0	0	0	0	0	0	0	0	0	0	0
RWC	0	0	0	0	0	0	0	0	0	0	0	1	0	0	0	0	0	0	0	0	0	0	0	0	0	0
Sci	1	2	0	0	0	0	0	0	5	0	0	0	0	2	0	0	0	0	2	0	0	2	0	0	0	2
Oil	0	1	0	2	1	0	0	0	0	0	0	0	0	0	1	2	0	0	0	0	0	0	0	0	1	0
CSI	0	0	0	0	0	0	0	2	0	0	0	0	0	0	0	0	0	0	0	0	0	0	0	0	0	0
TTr	0	0	0	0	0	0	0	0	0	0	0	1	0	0	0	0	0	0	0	0	0	0	0	0	0	0
TPn	1	0	0	0	0	1	0	0	0	0	0	0	0	0	0	0	0	0	0	0	0	0	0	0	0	0
SPn	0	0	0	0	0	0	0	0	0	0	0	0	0	0	0	0	0	0	1	0	0	0	0	0	1	1
STr	0	0	0	0	1	0	0	0	0	1	0	1	0	1	0	0	0	1	1	1	0	1	0	0	1	0
Fcc	0	0	0	0	0	0	0	2	0	0	0	0	0	0	0	0	0	0	0	0	0	0	0	0	0	0
Bcc	0	0	0	0	0	0	0	0	2	0	0	0	0	0	0	0	0	0	0	0	0	0	0	0	0	0
Scond	0	0	0	0	0	0	0	0	0	0	0	1	0	0	0	0	0	1	1	0	0	1	0	0	1	0
Tcond	0	0	0	0	0	0	0	0	0	0	0	1	0	0	0	0	0	0	0	0	0	2	0	0	0	0
Tci	0	0	0	1	0	0	0	0	0	0	1	0	0	0	0	0	0	0	0	0	0	2	0	0	0	0
Chl	0	0	0	0	0	0	0	0	0	0	0	0	1	0	0	0	3	0	0	0	0	0	1	1	1	1
QTLs associated with biotic stress tolerance																										
VR	4	0	0	0	0	2	0	0	2	0	2	0	0	2	0	16	0	0	0	0	0	0	31	0	0	0
FOV	0	0	0	0	0	0	0	0	0	0	0	0	0	1	0	0	1	0	1	0	1	0	0	2	1	0
CL	0	0	0	0	0	0	0	0	0	0	6	0	0	0	0	0	0	0	3	0	6	0	0	0	0	0

Table S2. Genomic positions of selected MQTL clusters and their associated candidate genes.

MQTL nomi	Right marker	Right marker position cM	Right marker position (bp)	Left marker	Left marker position (cM)	Left marker position (bp)	MQTL hududi (bp)	Nomzod gen ID
MQTLchr11-1	CIR0069	0	121 736 431 – 121 736 683	Gh256	0.4	122 260 644-122 260 744	122 208 313 - 122 286 960	LOC107944007, LOC107944005, LOC107898833, LOC121209554, LOC107944372, LOC107944370, LOC107944371, LOC107944373
MQTLchr14-1	CIR246	0	1 042 193 - 1 042 359	JESPR0156	2.32	2 043 731 - 2 043 815	1 400 586 - 1 564 644	LOC107938713, LOC107938721, LOC107938716, LOC121214898, LOC107938700, LOC107938696, LOC107938722, LOC107938702, LOC107938720, LOC107938698, LOC107938717, LOC107938714, LOC105796397, LOC107927504
MQTLchr17-1	HAU0912	85	50145014 - 50 145 235	NAU0855	87.74	53 365 517 - 53365735	52 254 939 – 52 501 783	LOC107918814, LOC107918720, LOC121215603, LOC107918618, LOC107918729, LOC107920503, LOC107918432, LOC107920171, LOC107920170, LOC107920169, LOC107920730, LOC107920729
MQTLchr19-2	NAU3012	19.35	2 961 543 - 2 961 724	NAU2233	20.21	3 116 626 - 3 116 869	2,996,763 – 3,070,813	LOC107907089, LOC121217522, LOC107907087, LOC107907085, LOC107907082, LOC107903281
MQTLchr23-1	TMB2901	41.8	32 011 368 - 32 011 548	MUSB1040	42.7	33 447 919 - 33 448 140	32,091,368 – 32,386,525	LOC107888944, LOC107892104, LOC107892107, LOC107892105, LOC107892108, LOC107892111, LOC107892112, LOC107892115,

								LOC107892113, LOC107892116, LOC107892118
MQTLchr24-1	NAU3804	46.67	53 520 610 - 53 520 782	BNL2616	47.96	54 410 660 - 54 410 807	53,520,610 – 54,410,807	LOC107900777, LOC107890566, LOC107923135, LOC107900777, LOC107899889, LOC107900771, LOC107900770, LOC107900768, LOC107900766, LOC107900765, LOC107900764, LOC107938339, LOC107938339, LOC107899887, LOC107900751, LOC107900744, LOC107900742, LOC107900739, LOC107900738, LOC107900737

Table S3. GO Enrichment Analysis of Molecular Functions, Biological Processes, and Cellular Components

Category	Enrichment FDR	Genes	Pathway Genes	Fold Enrichment	Pathway	Candidate genes
Biological function	0,0206	2	92	39,262	GO:0046274 Lignin catabolic process	LOC107944372, LOC107944371
Molecular function	0,0447	1	11	164,19	GO:0003984 Acetolactate synthase activity	LOC107938702
	0,0169	2	92	39,26	GO:0052716 Hydroquinone:oxygen oxidoreductase activity	LOC107944372, LOC107944371
	0,0169	3	307	17,65	GO:0050660 Flavin adenine dinucleotide binding	LOC107938702, LOC107907082, LOC107892112
	0,0447	2	215	16,8	GO:0005507 Copper ion binding	LOC107944372, LOC107944371
Cellular component	0,019	2	165	21,89	GO:0048046 Apoplast	LOC107944372, LOC107944371

Table S4. KEGG pathway enrichment analysis (FDR < 0.05)

FDR	$-\log_{10}$(FDR)	nGenes	Pathway Genes	Fold Enrichment	Pathway	Genes
0,005	2,3	2	37	102,5	Butanoate metabolism	LOC107938702<br>LOC107892107
0,01	2	2	95	39,9	Glyoxylate and dicarboxylate metabolism	LOC107892107<br>LOC107892112
0,012	1,92	3	378	15,1	Carbon metabolism	LOC107920503<br>LOC107892107<br>LOC107892112
0,016	1,8	2	130	29,9	2-Oxocarboxylic acid metabolism	LOC107938702<br>LOC107892112
0,021	1,68	2	163	23,3	Pyruvate metabolism	LOC107892107<br>LOC107892112
0,023	1,64	2	189	20,1	Glycolysis-Gluconeogenesis	LOC107892107<br>LOC107892112
0,028	1,55	1	15	126,4	C5-Branched dibasic acid metabolism	LOC107938702
0,028	1,55	1	16	118,5	Taurine and hypotaurine metabolism	LOC107907082
0,028	1,55	2	234	16,2	Endocytosis	LOC107920169<br>LOC107892102
0,044	1,36	2	344	11	Biosynthesis of amino acids	LOC107892112<br>LOC107938702

Figure S1. Distribution of QTLs across chromosomes in a consensus genetic map of cotton.

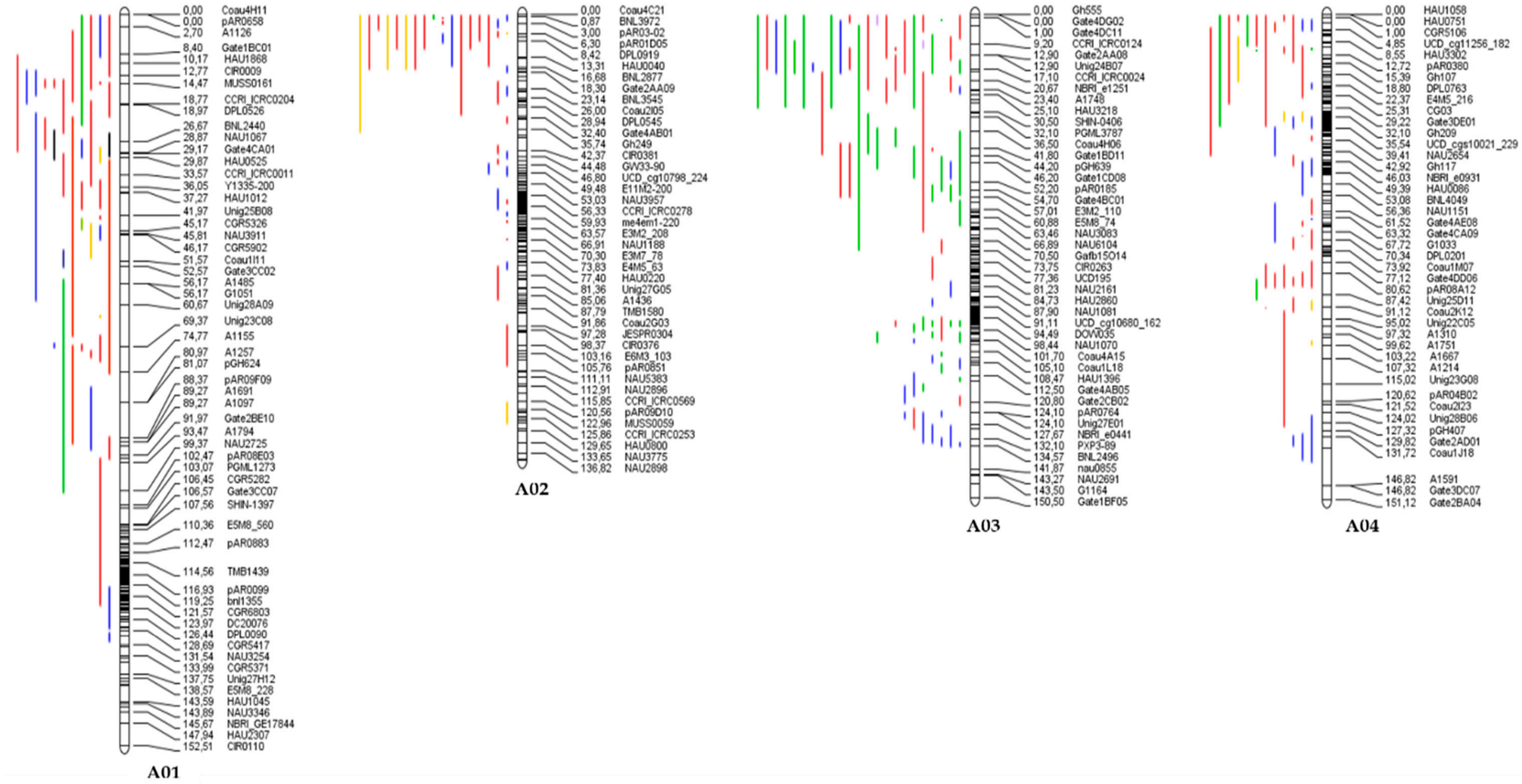

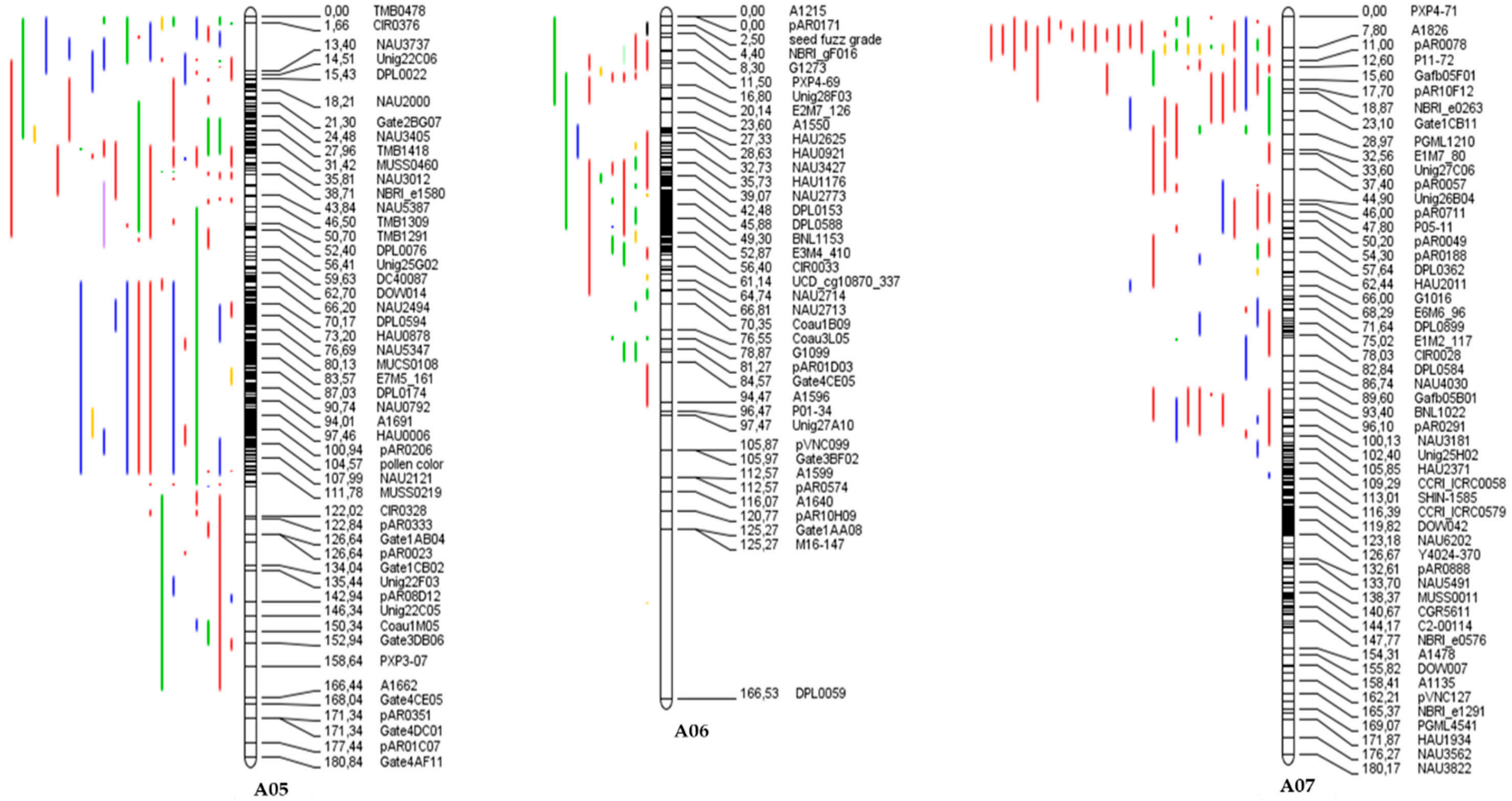

0,00 TMB0478
1,66 CIR0376
13,40 NAU3737
14,51 Unig22C06
15,43 DPL0022
18,21 NAU2000
21,30 Gate2BG07
24,48 NAU3405
27,96 TMB1418
31,42 MUSS0460
35,81 NAU3012
38,71 NBRI_e1580
43,84 NAU5387
46,50 TMB1309
50,70 TMB1291
52,40 DPL0076
56,41 Unig25G02
59,63 DC40087
62,70 DOW014
66,20 NAU2494
70,17 DPL0594
73,20 HAU0878
76,69 NAU5347
80,13 MUCS0108
83,57 E7M5_161
87,03 DPL0174
90,74 NAU0792
94,01 A1691
97,46 HAU0006
100,94 pAR0206
104,57 pollen color
107,99 NAU2121
111,78 MUSS0219
122,02 CIR0328
122,84 pAR0333
126,64 Gate1AB04
126,64 pAR0023
134,04 Gate1CB02
135,44 Unig22F03
142,94 pAR08D12
146,34 Unig22C05
150,34 Coau1M05
152,94 Gate3DB06
158,64 PXP3-07
166,44 A1662
168,04 Gate4CE05
171,34 pAR0351
171,34 Gate4DC01
177,44 pAR01C07
180,84 Gate4AF11
A05
0,00 A1215
0,00 pAR0171
2,50 seed fuzz grade
4,40 NBRI_gF016
8,30 G1273
11,50 PXP4-69
16,80 Unig28F03
20,14 E2M7_126
23,60 A1550
27,33 HAU2625
28,63 HAU0921
32,73 NAU3427
35,73 HAU1176
39,07 NAU2773
42,48 DPL0153
45,88 DPL0588
49,30 BNL1153
52,87 E3M4_410
56,40 CIR0033
61,14 UCD_cg10870_337
64,74 NAU2714
66,81 NAU2713
70,35 Coau1B09
76,55 Coau3L05
78,87 G1099
81,27 pAR01D03
84,57 Gate4CE05
94,47 A1596
96,47 P01-34
97,47 Unig27A10
105,87 pVNC099
105,97 Gate3BF02
112,57 A1599
112,57 pAR0574
116,07 A1640
120,77 pAR10H09
125,27 Gate1AA08
125,27 M16-147
166,53 DPL0059
A06
0,00 PXP4-71
7,80 A1826
11,00 pAR0078
12,60 P11-72
15,60 Gafb05F01
17,70 pAR10F12
18,87 NBRI_e0263
23,10 Gate1CB11
28,97 PGML1210
32,56 E1M7_80
33,60 Unig27C06
37,40 pAR0057
44,90 Unig26B04
46,00 pAR0711
47,80 P05-11
50,20 pAR0049
54,30 pAR0188
57,64 DPL0362
62,44 HAU2011
66,00 G1016
68,29 E6M6_96
71,64 DPL0899
75,02 E1M2_117
78,03 CIR0028
82,84 DPL0584
86,74 NAU4030
89,60 Gafb05B01
93,40 BNL1022
96,10 pAR0291
100,13 NAU3181
102,40 Unig25H02
105,85 HAU2371
109,29 CCRI_ICRC0058
113,01 SHIN-1585
116,39 CCRI_ICRC0579
119,82 DOW042
123,18 NAU6202
126,67 Y4024-370
132,61 pAR0888
133,70 NAU5491
138,37 MUSS0011
140,67 CGR5611
144,17 C2-00114
147,77 NBRI_e0576
154,31 A1478
155,82 DOW007
158,41 A1135
162,21 pVNC127
165,37 NBRI_e1291
169,07 PGML4541
171,87 HAU1934
176,27 NAU3562
180,17 NAU3822
A07

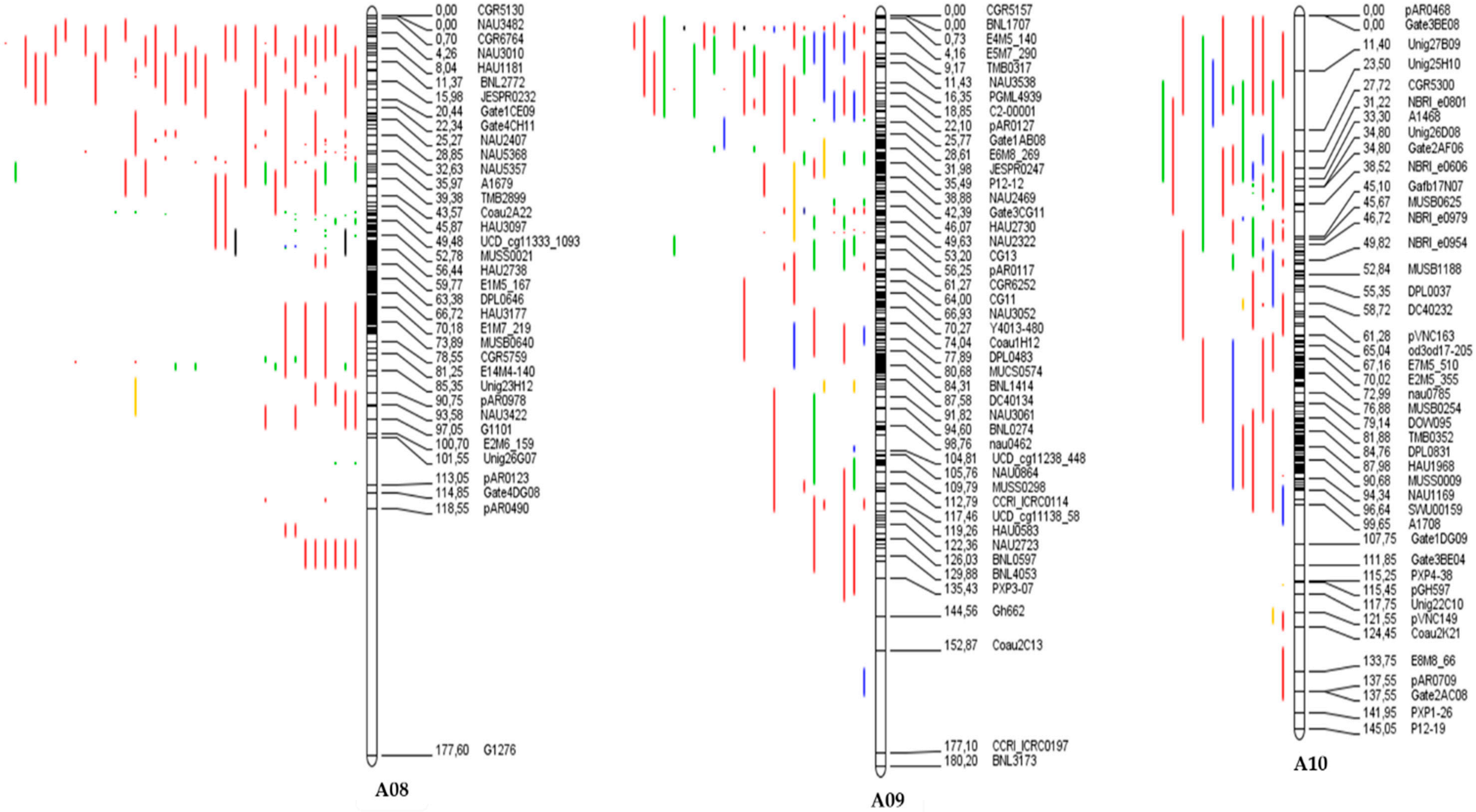
0,00 CGR5130
0,00 NAU3482
0,70 CGR6764
4,26 NAU3010
8,04 HAU1181
11,37 BNL2772
15,98 JESPR0232
20,44 Gate1CE09
22,34 Gate4CH11
25,27 NAU2407
28,85 NAU5368
32,63 NAU5357
35,97 A1679
39,38 TMB2899
43,57 Coau2A22
45,87 HAU3097
49,48 UCD_cg11333_1093
52,78 MUSS0021
56,44 HAU2738
59,77 E1M5_167
63,38 DPL0646
66,72 HAU3177
70,18 E1M7_219
73,89 MUSB0640
78,55 CGR5759
81,25 E14M4-140
85,35 Unig23H12
90,75 pAR0978
93,58 NAU3422
97,05 G1101
100,70 E2M6_159
101,55 Unig26G07
113,05 pAR0123
114,85 Gate4DG08
118,55 pAR0490
177,60 G1276
A08
0,00 CGR5157
0,00 BNL1707
0,73 E4M5_140
4,16 E5M7_290
9,17 TMB0317
11,43 NAU3538
16,35 PGML4939
18,85 C2-00001
22,10 pAR0127
25,77 Gate1AB08
28,61 E6M8_269
31,98 JESPR0247
35,49 P12-12
38,88 NAU2469
42,39 Gate3CG11
46,07 HAU2730
49,63 NAU2322
53,20 CG13
56,25 pAR0117
61,27 CGR6252
64,00 CG11
66,93 NAU3052
70,27 Y4013-480
74,04 Coau1H12
77,89 DPL0483
80,68 MUCS0574
84,31 BNL1414
87,58 DC40134
91,82 NAU3061
94,60 BNL0274
98,76 nau0462
104,81 UCD_cg11238_448
105,76 NAU0864
109,79 MUSS0298
112,79 CCRI_ICRC0114
117,46 UCD_cg11138_58
119,26 HAU0583
122,36 NAU2723
126,03 BNL0597
129,88 BNL4053
135,43 PXP3-07
144,56 Gh662
152,87 Coau2C13
177,10 CCRI_ICRC0197
180,20 BNL3173
A09
0,00 pAR0468
0,00 Gate3BE08
11,40 Unig27B09
23,50 Unig25H10
27,72 CGR5300
31,22 NBRI_e0801
33,30 A1468
34,80 Unig26D08
34,80 Gate2AF06
38,52 NBRI_e0606
45,10 Gafb17N07
45,67 MUSB0625
46,72 NBRI_e0979
49,82 NBRI_e0954
52,84 MUSB1188
55,35 DPL0037
58,72 DC40232
61,28 pVNC163
65,04 od3od17-205
67,16 E7M5_510
70,02 E2M5_355
72,99 nau0785
76,88 MUSB0254
79,14 DOW095
81,88 TMB0352
84,76 DPL0831
87,98 HAU1968
90,68 MUSS0009
94,34 NAU1169
96,64 SWU00159
99,65 A1708
107,75 Gate1DG09
111,85 Gate3BE04
115,25 PXP4-38
115,45 pGH597
117,75 Unig22C10
121,55 pVNC149
124,45 Coau2K21
133,75 E8M8_66
137,55 pAR0709
137,55 Gate2AC08
141,95 PXP1-26
145,05 P12-19
A10

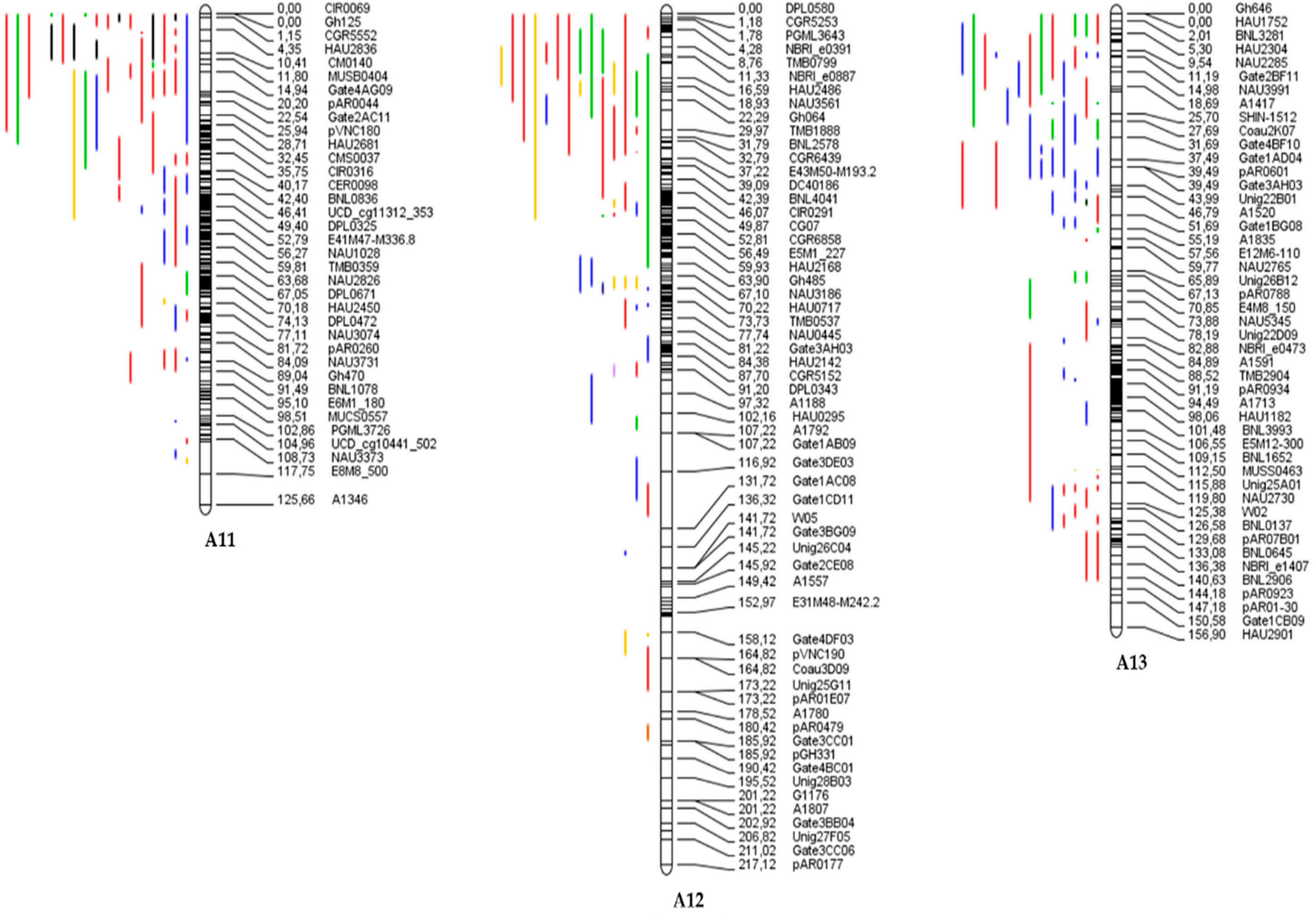
0,00 CIR0069
0,00 Gh125
1,15 CGR5552
4,35 HAU2836
10,41 CM0140
11,80 MUSB0404
14,94 Gate4AG09
20,20 pAR0044
22,54 Gate2AC11
25,94 pVNC180
28,71 HAU2681
32,45 CMS0037
35,75 CIR0316
40,17 CER0098
42,40 BNL0836
46,41 UCD_cg11312_353
49,40 DPL0325
52,79 E41M47-M336.8
56,27 NAU1028
59,81 TMB0359
63,68 NAU2826
67,05 DPL0671
70,18 HAU2450
74,13 DPL0472
77,11 NAU3074
81,72 pAR0260
84,09 NAU3731
89,04 Gh470
91,49 BNL1078
95,10 E6M1_180
98,51 MUCS0557
102,86 PGML3726
104,96 UCD_cg10441_502
108,73 NAU3373
117,75 E8M8_500
125,66 A1346
A11
0,00 DPL0580
1,18 CGR5253
1,78 PGML3643
4,28 NBRI_e0391
8,76 TMB0799
11,33 NBRI_e0887
16,59 HAU2486
18,93 NAU3561
22,29 Gh064
29,97 TMB1888
31,79 BNL2578
32,79 CGR6439
37,22 E43M50-M193.2
39,09 DC40186
42,39 BNL4041
46,07 CIR0291
49,87 CG07
52,81 CGR6858
56,49 E5M1_227
59,93 HAU2168
63,90 Gh485
67,10 NAU3186
70,22 HAU0717
73,73 TMB0537
77,74 NAU0445
81,22 Gate3AH03
84,38 HAU2142
87,70 CGR5152
91,20 DPL0343
97,32 A1188
102,16 HAU0295
107,22 A1792
107,22 Gate1AB09
116,92 Gate3DE03
131,72 Gate1AC08
136,32 Gate1CD11
141,72 W05
141,72 Gate3BG09
145,22 Unig26C04
145,92 Gate2CE08
149,42 A1557
152,97 E31M48-M242.2
158,12 Gate4DF03
164,82 pVNC190
164,82 Coau3D09
173,22 Unig25G11
173,22 pAR01E07
178,52 A1780
180,42 pAR0479
185,92 Gate3CC01
185,92 pGH331
190,42 Gate4BC01
195,52 Unig28B03
201,22 G1176
201,22 A1807
202,92 Gate3BB04
206,82 Unig27F05
211,02 Gate3CC06
217,12 pAR0177
A12
0,00 Gh646
0,00 HAU1752
2,01 BNL3281
5,30 HAU2304
9,54 NAU2285
11,19 Gate2BF11
14,98 NAU3991
18,69 A1417
25,70 SHIN-1512
27,69 Coau2K07
31,69 Gate4BF10
37,49 Gate1AD04
39,49 pAR0601
39,49 Gate3AH03
43,99 Unig22B01
46,79 A1520
51,69 Gate1BG08
55,19 A1835
57,56 E12M6-110
59,77 NAU2765
65,89 Unig26B12
67,13 pAR0788
70,85 E4M8_150
73,88 NAU5345
78,19 Unig22D09
82,88 NBRI_e0473
84,89 A1591
88,52 TMB2904
91,19 pAR0934
94,49 A1713
98,06 HAU1182
101,48 BNL3993
106,55 E5M12-300
109,15 BNL1652
112,50 MUSS0463
115,88 Unig25A01
119,80 NAU2730
125,38 W02
126,58 BNL0137
129,68 pAR07B01
133,08 BNL0645
136,38 NBRI_e1407
140,63 BNL2906
144,18 pAR0923
147,18 pAR01-30
150,58 Gate1CB09
156,90 HAU2901
A13

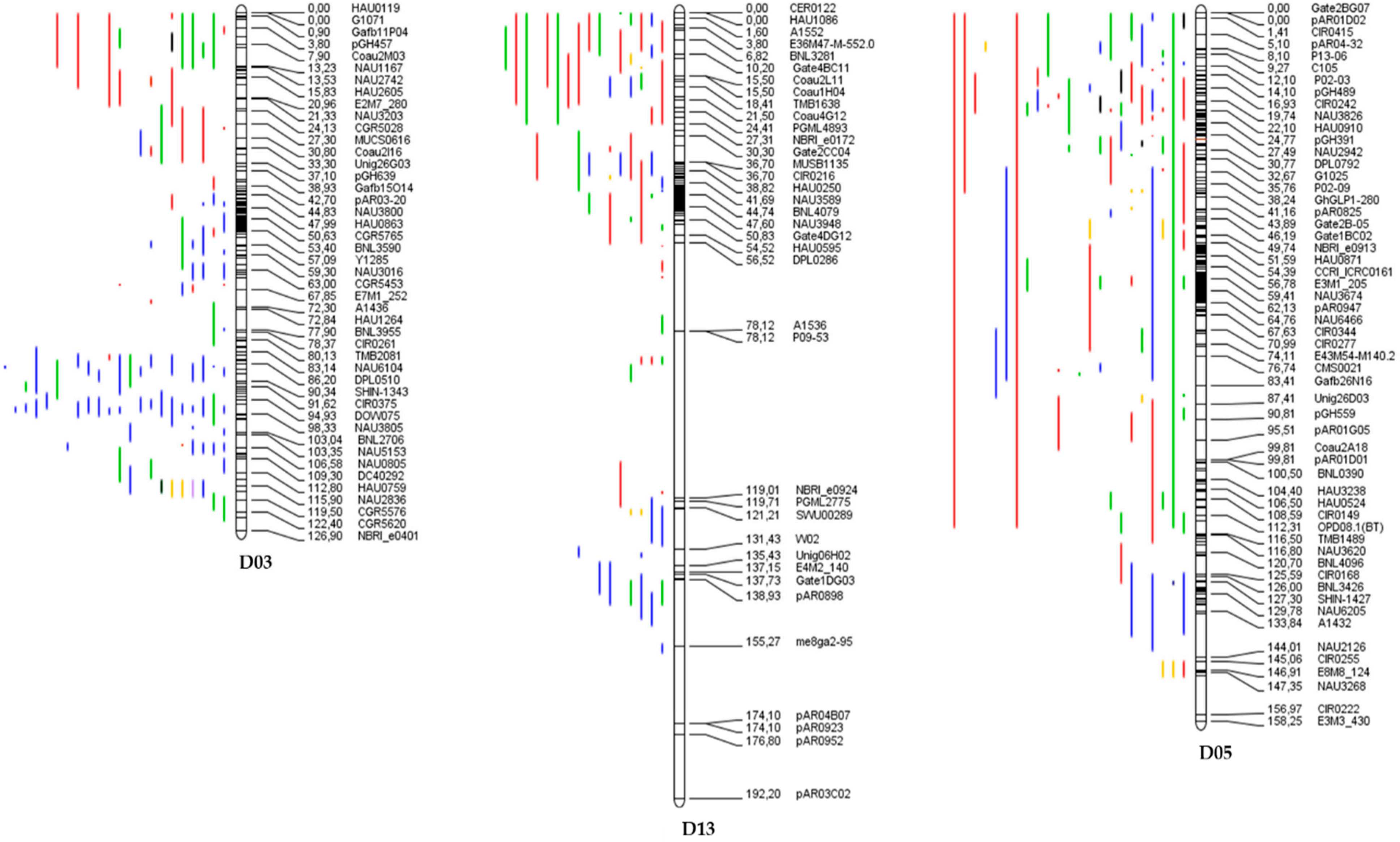

0,00 HAU0119
0,00 G1071
0,90 Gafb11P04
3,80 pGH457
7,90 Coau2M03
13,23 NAU1167
13,53 NAU2742
15,83 HAU2605
20,96 E2M7_280
21,33 NAU3203
24,13 CGR5028
27,30 MUCS0616
30,80 Coau2I16
33,30 Unig26G03
37,10 pGH639
38,93 Gafb15O14
42,70 pAR03-20
44,83 NAU3800
47,99 HAU0863
50,63 CGR5765
53,40 BNL3590
57,09 Y1285
59,30 NAU3016
63,00 CGR5453
67,85 E7M1_252
72,30 A1436
72,84 HAU1264
77,90 BNL3955
78,37 CIR0261
80,13 TMB2081
83,14 NAU6104
86,20 DPL0510
90,34 SHIN-1343
91,62 CIR0375
94,93 DOW075
98,33 NAU3805
103,04 BNL2706
103,35 NAU5153
106,58 NAU0805
109,30 DC40292
112,80 HAU0759
115,90 NAU2836
119,50 CGR5576
122,40 CGR5620
126,90 NBRI_e0401
D03
0,00 CER0122
0,00 HAU1086
1,60 A1552
3,80 E36M47-M-552.0
6,82 BNL3281
10,20 Gate4BC11
15,50 Coau2L11
15,50 Coau1H04
18,41 TMB1638
21,50 Coau4G12
24,41 PGML4893
27,31 NBRI_e0172
30,30 Gate2CC04
36,70 MUSB1135
36,70 CIR0216
38,82 HAU0250
41,69 NAU3589
44,74 BNL4079
47,60 NAU3948
50,83 Gate4DG12
54,52 HAU0595
56,52 DPL0286
78,12 A1536
78,12 P09-53
119,01 NBRI_e0924
119,71 PGML2775
121,21 SWU00289
131,43 W02
135,43 Unig06H02
137,15 E4M2_140
137,73 Gate1DG03
138,93 pAR0898
155,27 me8ga2-95
174,10 pAR04B07
174,10 pAR0923
176,80 pAR0952
192,20 pAR03C02
D13
0,00 Gate2BG07
0,00 pAR01D02
1,41 CIR0415
5,10 pAR04-32
8,10 P13-06
9,27 C105
12,10 P02-03
14,10 pGH489
16,93 CIR0242
19,74 NAU3826
22,10 HAU0910
24,77 pGH391
27,49 NAU2942
30,77 DPL0792
32,67 G1025
35,76 P02-09
38,24 GhGLP1-280
41,16 pAR0825
43,89 Gate2B-05
46,19 Gate1BC02
49,74 NBRI_e0913
51,59 HAU0871
54,39 CCRI_ICRC0161
56,78 E3M1_205
59,41 NAU3674
62,13 pAR0947
64,76 NAU6466
67,63 CIR0344
70,99 CIR0277
74,11 E43M54-M140.2
76,74 CMS0021
83,41 Gafb26N16
87,41 Unig26D03
90,81 pGH559
95,51 pAR01G05
99,81 Coau2A18
99,81 pAR01D01
100,50 BNL0390
104,40 HAU3238
106,50 HAU0524
108,59 CIR0149
112,31 OPD08.1(BT)
116,50 TMB1489
116,80 NAU3620
120,70 BNL4096
125,59 CIR0168
126,00 BNL3426
127,30 SHIN-1427
129,78 NAU6205
133,84 A1432
144,01 NAU2126
145,06 CIR0255
146,91 E8M8_124
147,35 NAU3268
156,97 CIR0222
158,25 E3M3_430
D05

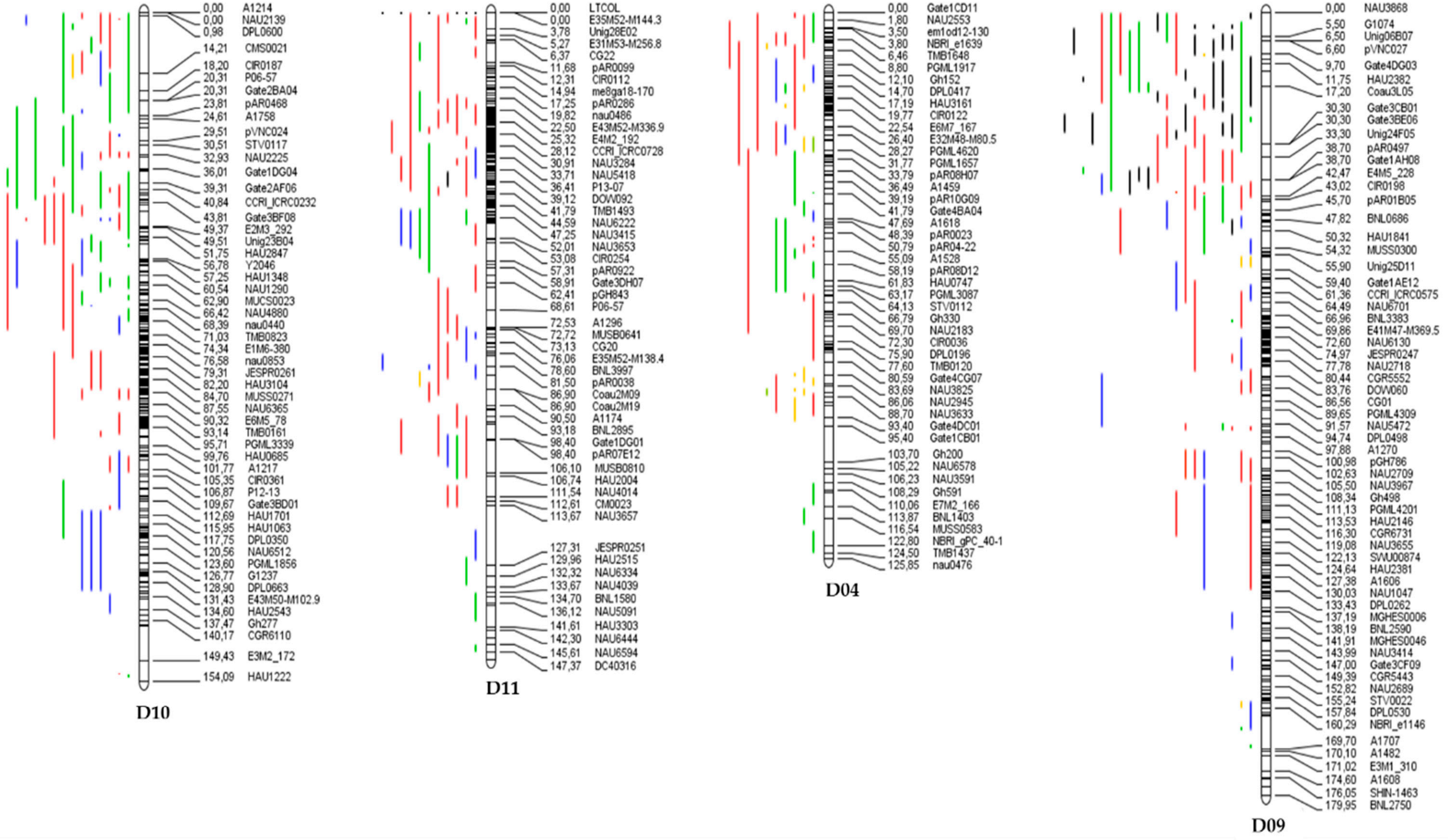
0,00 A1214
0,00 NAU2139
0,98 DPL0600
14,21 CMS0021
18,20 CIR0187
20,31 P06-57
20,31 Gate2BA04
23,81 pAR0468
24,61 A1758
29,51 pVNC024
30,51 STV0117
32,93 NAU2225
36,01 Gate1DG04
39,31 Gate2AF06
40,84 CCRI_ICRC0232
43,81 Gate3BF08
49,37 E2M3_292
49,51 Unig23B04
51,75 HAU2847
56,78 Y2046
57,25 HAU1348
60,54 NAU1290
62,90 MUCS0023
66,42 NAU4880
68,39 nau0440
71,03 TMB0823
74,34 E1M6-380
76,58 nau0853
79,31 JESPR0261
82,20 HAU3104
84,70 MUSS0271
87,55 NAU6365
90,32 E6M5_78
93,14 TMB0161
95,71 PGML3339
99,76 HAU0685
101,77 A1217
105,35 CIR0361
106,87 P12-13
109,67 Gate3BD01
112,69 HAU1701
115,95 HAU1063
117,75 DPL0350
120,56 NAU6512
123,60 PGML1856
126,77 G1237
128,90 DPL0663
131,43 E43M50-M102.9
134,60 HAU2543
137,47 Gh277
140,17 CGR6110
149,43 E3M2_172
154,09 HAU1222
D10
0,00 LTCOL
0,00 E35M52-M144.3
3,78 Unig28E02
5,27 E31M53-M256.8
6,37 CG22
11,68 pAR0099
12,31 CIR0112
14,94 me8ga18-170
17,25 pAR0286
19,82 nau0486
22,50 E43M52-M336.9
25,32 E4M2_192
28,12 CCRI_ICRC0728
30,91 NAU3284
33,71 NAU5418
36,41 P13-07
39,12 DOW092
41,79 TMB1493
44,59 NAU6222
47,25 NAU3415
52,01 NAU3653
53,08 CIR0254
57,31 pAR0922
58,91 Gate3DH07
62,41 pGH843
68,61 P06-57
72,53 A1296
72,72 MUSB0641
73,13 CG20
76,06 E35M52-M138.4
78,60 BNL3997
81,50 pAR0038
86,90 Coau2M09
86,90 Coau2M19
90,50 A1174
93,18 BNL2895
98,40 Gate1DG01
98,40 pAR07E12
106,10 MUSB0810
106,74 HAU2004
111,54 NAU4014
112,61 CM0023
113,67 NAU3657
127,31 JESPR0251
129,96 HAU2515
132,32 NAU6334
133,67 NAU4039
134,70 BNL1580
136,12 NAU5091
141,61 HAU3303
142,30 NAU6444
145,61 NAU6594
147,37 DC40316
D11
0,00 Gate1CD11
1,80 NAU2553
3,50 em1od12-130
3,80 NBRI_e1639
6,46 TMB1648
8,80 PGML1917
12,10 Gh152
14,70 DPL0417
17,19 HAU3161
19,77 CIR0122
22,54 E6M7_167
26,40 E32M48-M80.5
28,27 PGML4620
31,77 PGML1657
33,79 pAR08H07
36,49 A1459
39,19 pAR10G09
41,79 Gate4BA04
47,69 A1618
48,39 pAR0023
50,79 pAR04-22
55,09 A1528
58,19 pAR08D12
61,83 HAU0747
63,17 PGML3087
64,13 STV0112
66,79 Gh330
69,70 NAU2183
72,30 CIR0036
75,90 DPL0196
77,60 TMB0120
80,59 Gate4CG07
83,69 NAU3825
86,06 NAU2945
88,70 NAU3633
93,40 Gate4DC01
95,40 Gate1CB01
103,70 Gh200
105,22 NAU6578
106,23 NAU3591
108,29 Gh591
110,06 E7M2_166
113,87 BNL1403
116,54 MUSS0583
122,80 NBRI_gPC_40-1
124,50 TMB1437
125,85 nau0476
D04
0,00 NAU3868
5,50 G1074
6,50 Unig06B07
6,60 pVNC027
9,70 Gate4DG03
11,75 HAU2382
17,20 Coau3L05
30,30 Gate3CB01
30,30 Gate3BE06
33,30 Unig24F05
38,70 pAR0497
38,70 Gate1AH08
42,47 E4M5_228
43,02 CIR0198
45,70 pAR01B05
47,82 BNL0686
50,32 HAU1841
54,32 MUSS0300
55,90 Unig25D11
59,40 Gate1AE12
61,36 CCRI_ICRC0575
64,49 NAU6701
66,96 BNL3383
69,86 E41M47-M369.5
72,60 NAU6130
74,97 JESPR0247
77,78 NAU2718
80,44 CGR5552
83,76 DOW060
86,56 CG01
89,65 PGML4309
91,57 NAU5472
94,74 DPL0498
97,88 A1270
100,98 pGH786
102,63 NAU2709
105,50 NAU3967
108,34 Gh498
111,13 PGML4201
113,53 HAU2146
116,30 CGR6731
119,08 NAU3655
122,13 SWU00874
124,64 HAU2381
127,38 A1606
130,03 NAU1047
133,43 DPL0262
137,19 MGHES0006
138,19 BNL2590
141,91 MGHES0046
143,99 NAU3414
147,00 Gate3CF09
149,39 CGR5443
152,82 NAU2689
155,24 STV0022
157,84 DPL0530
160,29 NBRI_e1146
169,70 A1707
170,10 A1482
171,02 E3M1_310
174,60 A1608
176,05 SHIN-1463
179,95 BNL2750
D09

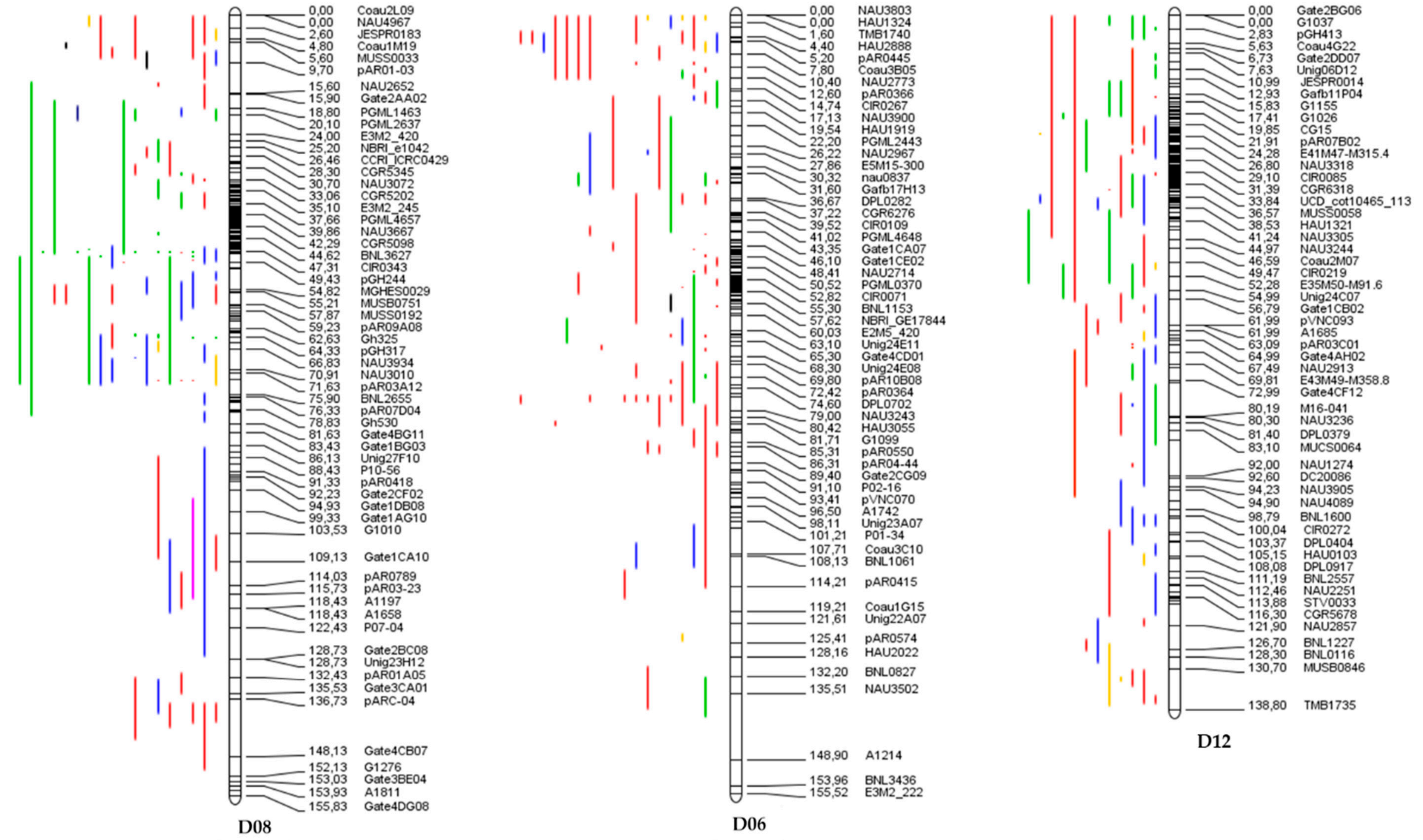

Figure S1 Distribution of QTLs across chromosomes in a consensus genetic map of cotton. QTLs related to fiber quality traits are depicted in red, yield-related traits in green, morpho-biological traits in blue, biotic stress-related traits in black, and biochemical and physiological traits are represented by yellow lines.
